# A Multi-Scale Densely Connected Convolutional Neural Network for Automated Thyroid Nodule Classification

**DOI:** 10.3389/fnins.2022.878718

**Published:** 2022-05-19

**Authors:** Luoyan Wang, Xiaogen Zhou, Xingqing Nie, Xingtao Lin, Jing Li, Haonan Zheng, Ensheng Xue, Shun Chen, Cong Chen, Min Du, Tong Tong, Qinquan Gao, Meijuan Zheng

**Affiliations:** ^1^College of Physics and Information Engineering, Fuzhou University, Fuzhou, China; ^2^Fujian Key Lab of Medical Instrumentation & Pharmaceutical Technology, Fuzhou University, Fuzhou, China; ^3^Fujian Medical University Union Hospital, Fuzhou, China; ^4^Fujian Medical Ultrasound Research Institute, Fuzhou, China

**Keywords:** the thyroid nodule classification, multi-scale, densely connection, hybrid atrous convolution, deep convolutional neural network

## Abstract

Automated thyroid nodule classification in ultrasound images is an important way to detect thyroid nodules and to make a more accurate diagnosis. In this paper, we propose a novel deep convolutional neural network (CNN) model, called n-ClsNet, for thyroid nodule classification. Our model consists of a multi-scale classification layer, multiple skip blocks, and a hybrid atrous convolution (HAC) block. The multi-scale classification layer first obtains multi-scale feature maps in order to make full use of image features. After that, each skip-block propagates information at different scales to learn multi-scale features for image classification. Finally, the HAC block is used to replace the downpooling layer so that the spatial information can be fully learned. We have evaluated our n-ClsNet model on the TNUI-2021 dataset. The proposed n-ClsNet achieves an average accuracy (ACC) score of 93.8% in the thyroid nodule classification task, which outperforms several representative state-of-the-art classification methods.

## 1. Introduction

Proper balancing of hormones, which regulates metabolism in the human body, is a main sign to identify the healthy nature of human beings. The tyroid gland is responsible for balancing hormones in human being. Therefore, the thyroid is an essential butterfly shaped organ which is positioned in front of the neck (Gulame et al., 2021). A thyroid nodule is a discrete lesion within the thyroid gland that is radiologically distinct from the surrounding thyroid parenchyma (Haugen et al., 2016). Thyroid nodules are very common in the general population. About 19–68% of individuals are detected to have thyroid nodules with high resolution ultrasound imaging (Liu et al., 2019). Generally, only nodules >1 cm should be evaluated, since they have a greater potential to be clinically significant cancers. In very rare cases, some nodules <1 cm yet may cause future morbidity and mortality Haugen et al. (2016). Thyroid cancer accounts for 3% of the global incidence of all cancers, with 586,000 new patients estimated in Miranda-Filho et al. (2021). Since ultrasound image provides a non-invasive and realtime inspection at a low cost, ultrasonography has become the best selection for the clinical identification of thyroid nodules (Gulame et al., 2021). However, due to ultrasound image being influenced by echo and speckle noise, experienced radiologists usually diagnose based on the shape, margin, and boundaries of sonographic characteristics of nodules in ultrasound image slices. It is fairly subjective and extremely dependent upon the clinical experience of radiologists (Yang et al., 2021). So as to handle this challenge, computer aided classification using ultrasound images is quite important in thyroid nodule identification. The automatic classification of thyroid nodules can differentiate whether a nodule is benign or malignant, which reduces the workload and inexperienced young radiologists' misdiagnosis rate (Yang et al., 2021).

Two steps are required in machine learning (ML)-based methods for thyroid nodule classification. Features are first extracted and then a classifier is built to perform an automated classification. For instance, random forest Ouyang et al. (2019), backpropagation neural network (BPNN) Kumari and Rani (2019), and stationary wavelet transform Acharya et al. (2014) have been well applied in the classification of thyroid nodules.

In recent years, deep learning models have successfully been used in image classification tasks, since they have shown superior performance to conventional learning methods. One benefit of deep learning is that it can extract deep features hidden in the sonographic image that human radiologists may not visually inspect. In addition, it can integrate the feature extraction and classification into a uniform framework, which avoids the processes of complex hand-crafted features extraction and classifier selection (Yang et al., 2021). Therefore, various deep learning-based methods have been proposed for different classification tasks.

For the classification of natural images, Krizhevsky et al. (2012) presented a groundbreaking networks, which demonstrated that deep learning models have superior performance in the classification domain. Szegedy et al. (2015) used a deep convolutional neural network (CNN) architecture called Inception, which obtained further improvements in image classification over AlexNet (Krizhevsky et al., 2012). Simonyan and Zisserman (2014) proposed a very deep convolutional network, which moved a step forward to deepen networks in image recognition. He et al. (2016) proposed an extraordinary structure referred to as ResNet, which solved the degradation problem in an extremely deep convolutional network. Iandola et al. (2016) proposed a lightweight CNN architecture called SqueezeNet to speed up the inference process without loosing accuracy (ACC). Howard et al. (2017) presented a lightweight and efficient neural network, which can achieve a high ACC of classification. Huang et al. (2017) introduced the dense convolutional network to strengthen feature propagation and encourage feature reuse.

Several studies based on deep neural networks have been carried out for the classification of thyroid nodules (Song et al., 2018; Zhang et al., 2019a). However, the use of classification models in a natural image may lead to poor generalization problems. First of all, since the amount of natural images is much larger than the number of medical images, it is difficult to achieve the same ACC for the classification of thyroid nodules on the Caltech-101 dataset. Different from natural images, it is difficult to obtain millions of ultrasound images in clinical practice. Therefore, it is a challenge to train deep learning models using a small set of ultrasound images for the classification of thyroid nodules. In clinical practice, experienced radiologists distinguish whether a thyroid nodule is benign or malignant in ultrasound image slices *via* visual inspections. However, the process is not only time consuming and has high labor cost, but also has extremely subjective biases.

Based on the advantages of CNN and Transformer, we propose an O-Net framework to combine the CNN and the Transformer to learn both global and local contextual features. We combine the CNN and Swin Transformer as encoder first and send them into a CNN-based decoder and a Swin Transformer-based decoder, respectively. The results of two decoders are fused to get the final result. This network combines the advantages of CNN and Transformer and may improve the performance of medical image segmentation. Our experimental results have shown that the performance of the network can be significantly improved by combining CNN and Transformer. In addition, a classification task is simultaneously performed based on the O-Net. Experiments show that the segmentation results are beneficial for improving the ACC of the classification task. Experiments on the Synapse multi-organ segmentation dataset and the ISIC2017 skin lesion challenge dataset have demonstrated the superiority of our method compared to other state-of-the-art segmentation methods. In addition, based on the segmentation network, the performance of the classification network has also been greatly improved.

Data is the key to the performance of classification networks based on deep learning. Classification networks that show good performance in natural images are difficult to achieve the same high ACC in medical image classification. Most of the existing thyroid nodule classification methods use the natural image classification network as the backbone network architecture. However, the classification network used for natural images does not fully adapt to medical images because the number of thyroid nodule images is far less than that of natural images. In the case of small amount of data, there is a risk of overfitting when deep network suitable for natural images is used to classify thyroid nodules. Therefore, this paper proposes a new method to solve this problem.

The method includes the following steps. First, we adopt a multi-scale input layer to excavate multiscale features. Then, we design specialized skip-block exploit depth features. Finally, we employ hybrid atrous convolution block substitute downsampling. In general, the main innovations of this paper include the follwing:

1) We design a skip-block as depth feature extractor, which consists of convolution layer, batch normalization layer, skip connection layer, and activation function. This skip-block is used to learn the deep features of thyroid nodules. Its skip connection structure deepens the network while reducing the risk of overfitting.

2) We propose a novel hybrid atrous convolution (HAC) block substitute downsampling in order to reduce the loss of spatial information caused by downsampling. This framework with HAC effectively enlarges the receptive fields of the network to aggregate global information.

## 2. Related Works

### 2.1. Thyroid Nodule Classification Based on ML

Computer-aided diagnostic (CAD) system of thyroid nodules has a long history. For objective differentiation of benign/malignant thyroid lesions, various CAD systems based on ML have been exploited (Chang et al., 2010, 2016; Iakovidis et al., 2010; Acharya et al., 2011, 2012; Ding et al., 2011; Raghavendra et al., 2017; Ardakani et al., 2018; Prochazka et al., 2019a,b; Lu et al., 2020).

Earlier ML approaches for thyroid nodule classification include two steps: hand-crafted features are first extracted and then used in the support vector machine (SVM) or k-nearest neighbor (KNN) classifier to build the automated classification system for the diagnosis of malignant thyroid nodules (Chang et al., 2010; Iakovidis et al., 2010; Acharya et al., 2011; Ding et al., 2011).

Afterward, the CAD system used for thyroid nodules tries to consider combining various features and different classifiers. In another study by Acharya et al. (2012), integrated features include the following: local binary pattern, laws texture energy, Fourier descriptor, and Fourier spectrum descriptor, using ultrasound images of 20 nodules (10 benign images and 10 malignant images) to extract features. Then, resulting feature vectors were used to build seven different classifiers in order to compare the performances, including SVM, decision tree, sugeno fuzzy, gaussian mixture model (GMM), KNN, radial basis probabilistic neural network, and naive Bayes classifier. The result shows that SVM and fuzzy classifier achieved the highest classification ACC of 100%, whereas, the GMM classifier peaked at an ACC of 98%. Chang et al. (2016) employed histogram, intensity differences, elliptical fit, gray-level co-occurrence matrices, and gray-level run-length matrices to abstract features from 30 malignant and 29 benign images, which then used SVM classifier and leaveone-out cross-validation to differentiate benign and malignant nodules, consequently achieving ACC of 98.4%. Raghavendra et al. (2017) proposed the CAD based on a binary stack decomposition algorithm, which extracted 181 features from 242 images and achieved ACC of 97.5% using SVM classifier. Therefore, we can conclude that selecting features and then constructing a classifier is very important for thyroid nodule classification, which is the key to promoting classification ACC.

From more recently published studies, how to extract more features from the original image and select features carefully is still the key to thyroid nodule classification based on ML. Ardakani et al. (2018) proposed the CAD based on textural and morphological features, which is capable of distinguishing thyroid nodules from ultrasound images by utilizing a support vector machine classifier. Prochazka et al. (2019b) designed a CAD that divided 60 thyroid nodules (20 malignant images, 40 benign images) into small patches of 17 × 17 pixels, which were used to extract several direction independent features by employing two-threshold binary decomposition. The features were used in random forests (RF) and SVM classifiers to categorize nodules into malignant and benign classes, then obtained the ACC score of 91.6%. In another study, Prochazka et al. (2019a) applied histogram analysis and segmentation-based fractal texture analysis algorithm, which calculated direction-independent features only. The features were used in SVM and RF classifiers to differentiate nodules into malignant and benign classes. Using the leave-one-out cross-validation method, the overall ACC was 92.42% for RF and 94.64% for SVM. Lu et al. (2020) extracted shape features, texture features, and local binary pattern features from original ultrasound images (59 patients). Then, the multi-kernel support vector machine classifier was configured with 10 linear kernels to combine features from different categories for classification, achieving the best ACC for the sub-class at 94.44%.

### 2.2. Thyroid Nodule Classification Based on Deep Learning

The classical ML algorithms usually require complex feature engineering, which first selects features and then uses it in classifier. However, the deep learning only needs to pass the data directly to the neural networks. Thus, one of the most growing trends of ML is deep learning (Sharifi et al., 2021). Song et al. (2018) developed a cascade convolution neural network framework, which confirmed the feasibility of CNN used for thyroid nodules detection and recognition. Zhang et al. (2019a) adopted a tripartite classification module based on CNN model to pick out nodules information in ultrasound images. Wang et al. (2020) proposed a dual-attention ResNet-based classification network to automatically achieve the accurate classification of thyroid nodules. Specifically, they adopted ResNet200 as the backbone network architecture to perform the classification of thyroid nodules while there is the problem that the classification network used for natural images does not fully adapt to medical images.

## 3. The Proposed Method

### 3.1. Overall Architecture Design

**Figure 1:**
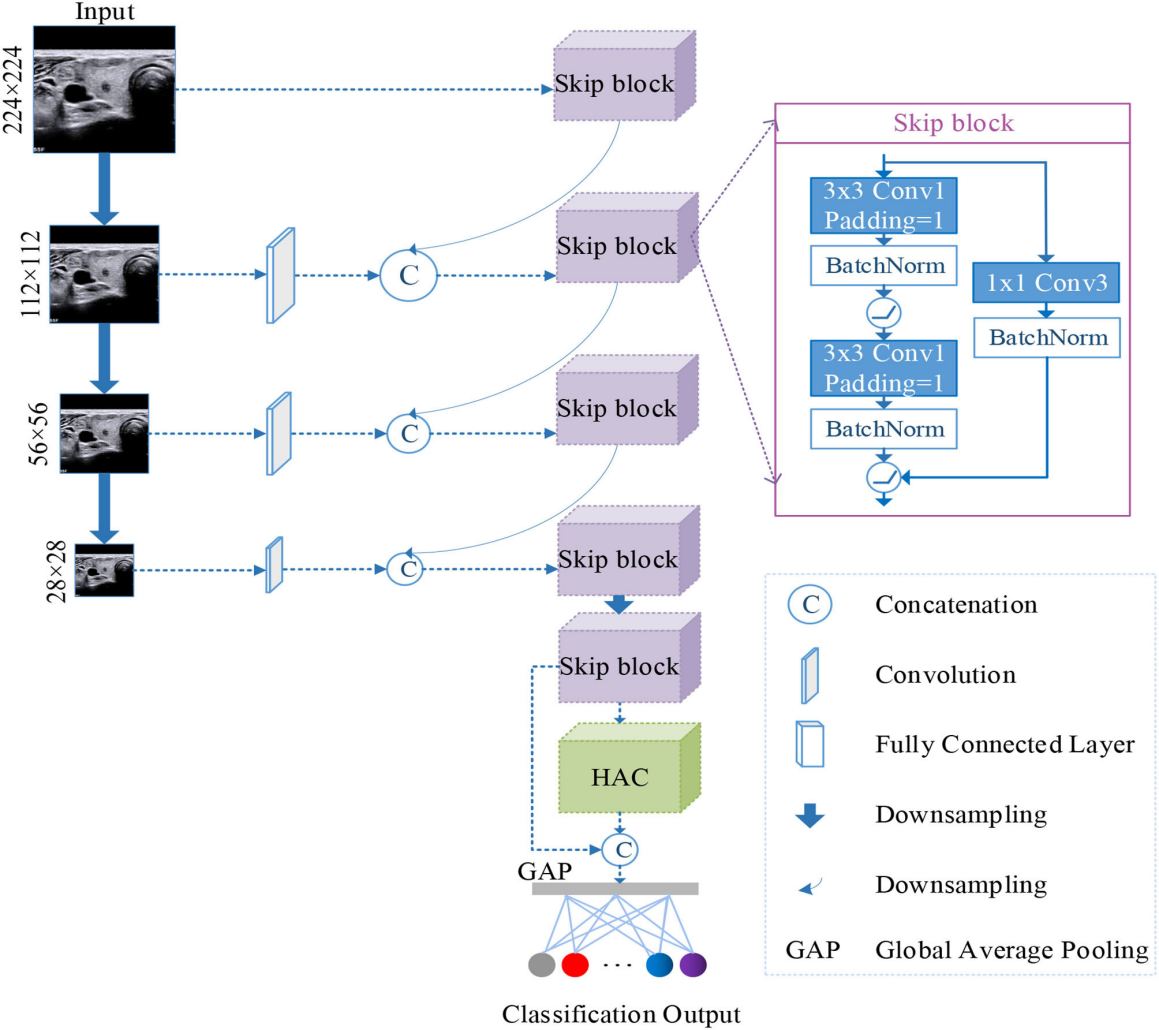
Illustration of the architecture of the proposed n-ClsNet network, which consists of two blocks: Skip-block and hybrid atrous convolution (HAC) block.

In this paper, we proposed a n-ClsNet classification model, which consists of a multi-scale classification layer, skip blocks, and HAC block. The multi-scale classification layer supported the n-ClsNet model capture several scale features on small-scale dataset. In an insufficient data case, tackled various features are quite important to classification networks. In each skip block, the convolution with skip connection can handle several scale information from a multiscale layer. Our proposed n-ClsNet is specialized for benign and malignant binary classification tasks of thyroid nodules. The n-ClsNet network's framework is shown in Figure 1.

### 3.2. Image Preprocess

**Figure 2:**
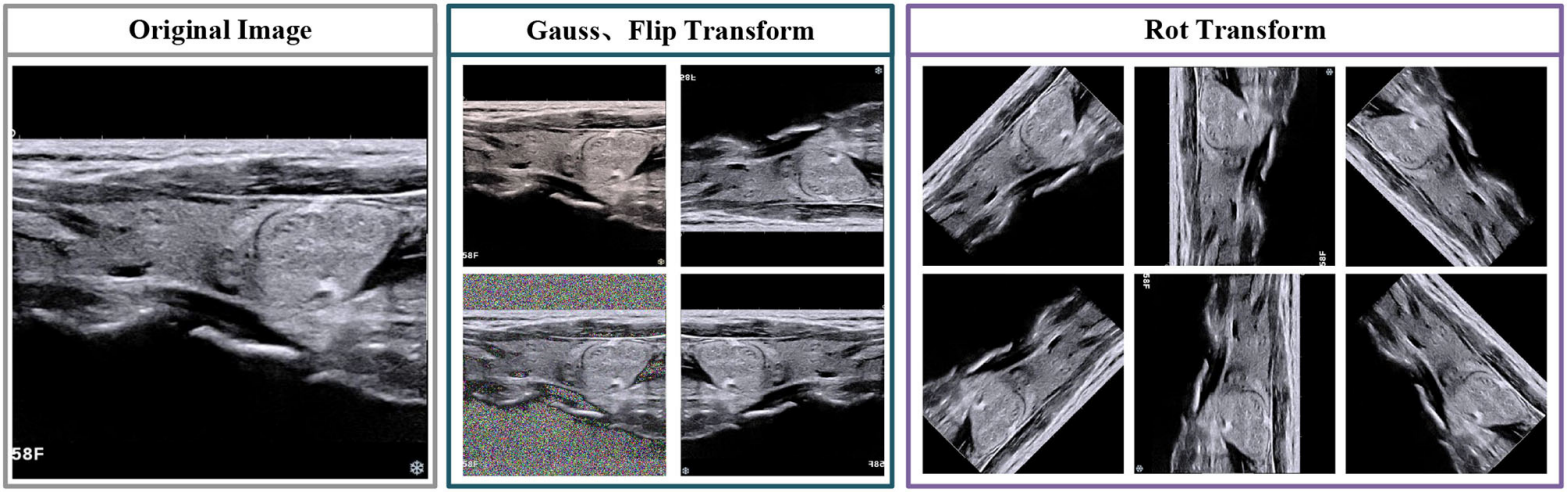
Examples of several transformations for thyroid nodule image.

In this paragraph, we foremost introduce the data augmentation and image pretreatment strategies, which are used in training and testing stages. Due to the limited number of medical image datasets, the datasets are enlarged to reduce the hazard of overfitting (Sun et al., 2018). For image preprocessing, we first select several transformations, includes vertical flip, horizontal flip and rotation. The direction of rotation including 45/90/135/180/225/270/315. Besides, we employ noise interference, which selected gauss noise. The visualization of transformation is shown in Figure 2.

### 3.3. Multi-Scale Classification Framework

**Figure 3:**
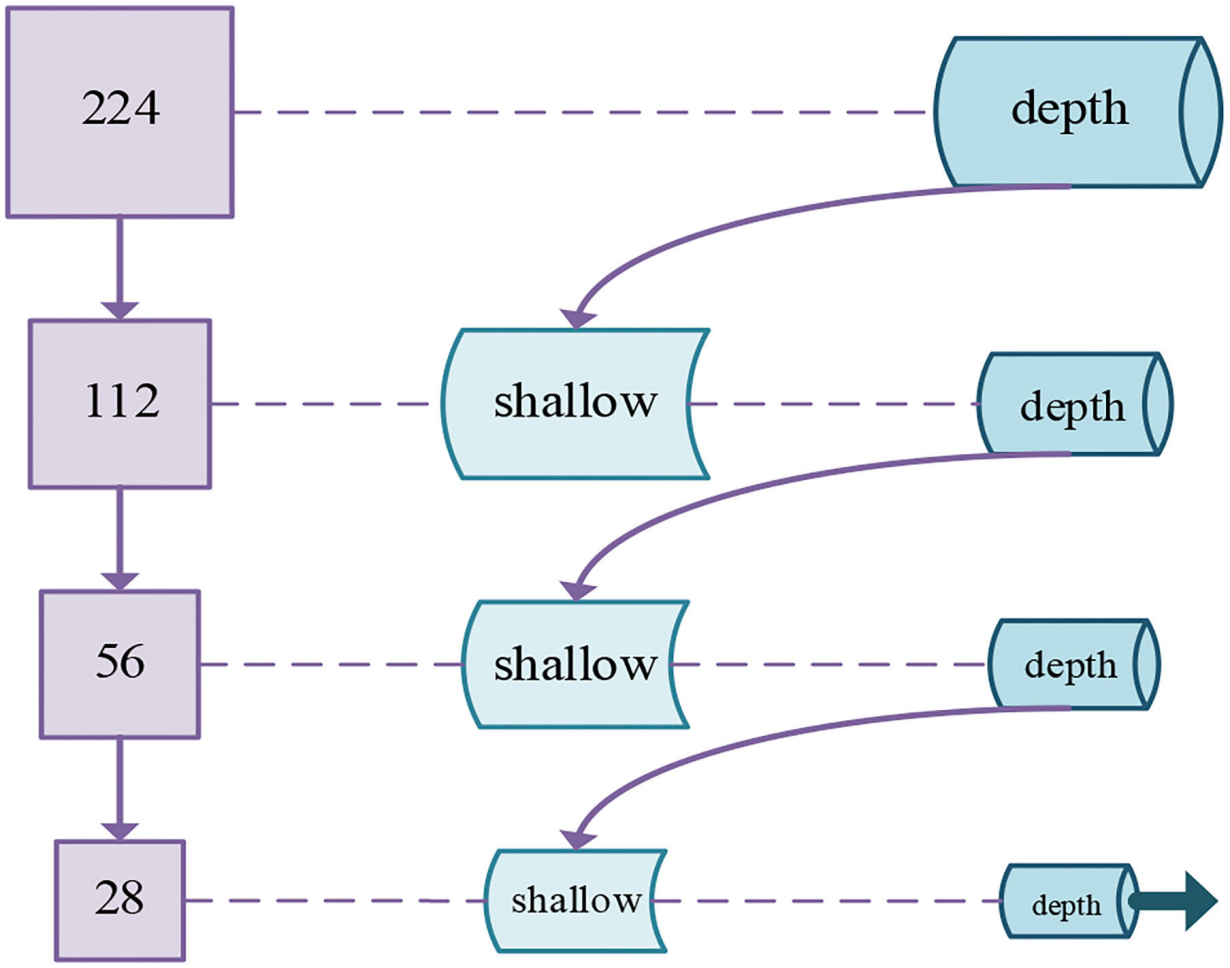
Schematic diagram of our proposed multi-scale classification framework for thyroid nodules.

The multi-scale input layer was extensively used for the segmentation of images. The deep learning model that adopted multi-scale input has been demonstrated to increase the performance of segmentation (Gu et al., 2019). Because multi-scale input could integrate various information from feature maps to avoid the large parameters in follow-up networks. Not only that, multi-scale input could enlarge the network width. Therefore, the above advantages of multi-scale input can be applied not only to image segmentation algorithms but also to image classification algorithms. Then, we determine to introduce a multi-scale method into the n-ClsNet to achieve supplementary feature representation in scale interspace. Different from Fu et al. (2018), they pushed the multiscale feature map to multi-scream networks and concatenate the ultimate feature map in the last layer, we employ the max-pooling layer to downsampling the image effectively and construct the feature detectors with different receptive field sizes. In our multi-scale classification framework, we first adopt downsampling of different multiples in order to obtain image patches sample of different sizes. According to the size of the original image from thyroid nodules, we design four branches downsampling with four scales. In each branch, the thyroid nodules image is followed by feature extractors in order that receiving abundant information on image features. Then, each branch connects to the former branch. Specifically, the first branch only has a depth feature extractor while others have both shallow feature extractor and depth feature extractor. The shallow feature extractor from the last three branches was combined with the depth feature extractor from the former branch. This method is more advantageous for characterizing diverse size structures in ultrasound imaging than single scale framework. The architecture of this multi-scale classification framework is shown in Figure 3.

### 3.4. Skip Block

**Figure 4:**
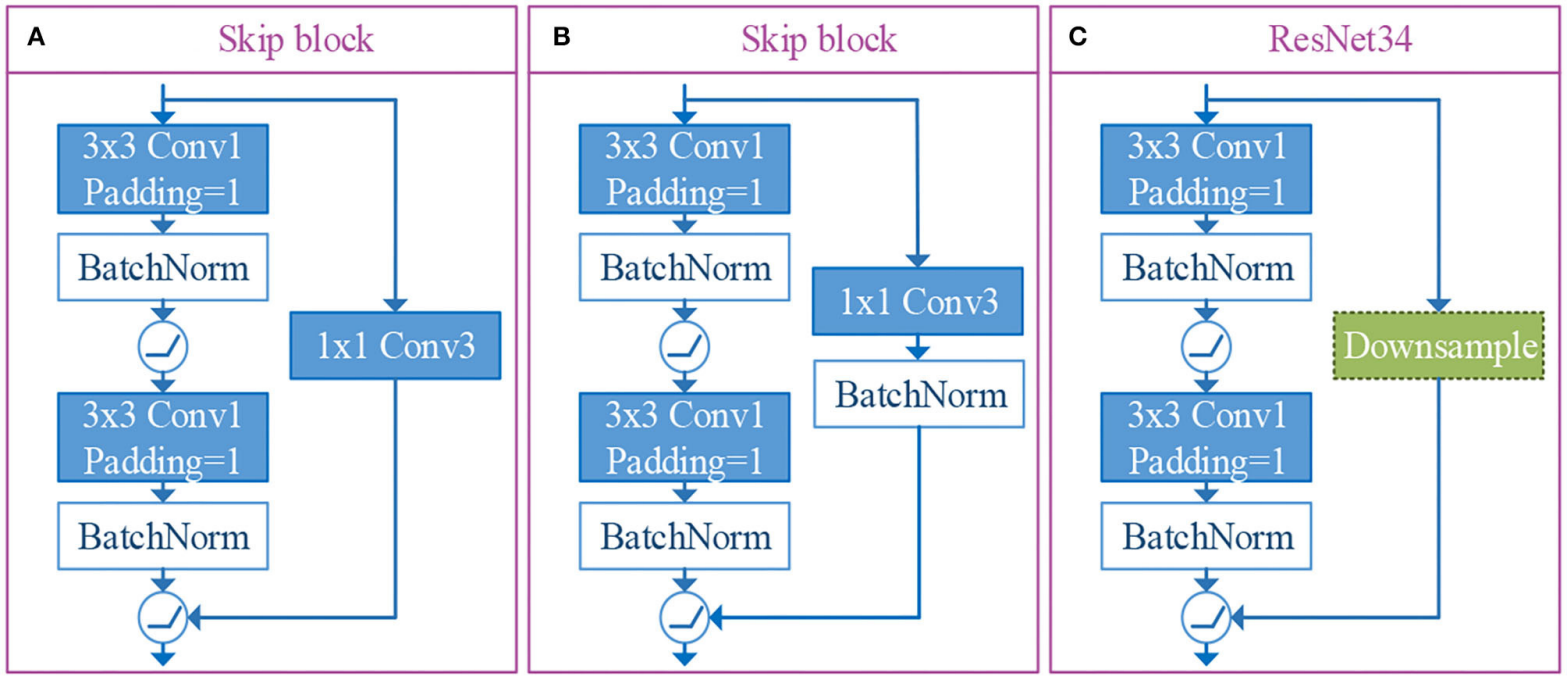
The illustrations of skip connection layer and residual architecture of resnet34-layer. Among them, **(A, B)** are the versions of skip connection layer designed by us, and **(C)** is the residual architecture of ResNet34-layer.

In our n-ClsNet framework, we design the skip-block as a depth feature extractor, which receives shallow feature map from the former convolution layer. This skip-block is composed of a convolution layer, batch normalization layer, skip connection layer, and activation function. With regard to convolution layer from skip-block, we select 3 × 3 convolutional kernels and stuff a layer of edge pixels. They are followed by a batch normalization layer in order to alleviate the disappearance of gradients. After that, the ReLU is used as the activation function, which introduced a non-linear element to further overcome the problem of vanishing gradient. The skip connection layer is designed to leapfrog the structure composed of the convolutional layer, batch normalization layer, and ReLU. This layer is directly connected to a straight link that pushed the output of the shallow feature extractor and pushed into the ReLU. In order to conform to the demand of thyroid nodules datasets, we tried two kinds of skip connection layers and compared the residual architecture of the ResNet34-layer (Simonyan and Zisserman, 2014). This residual architecture is shown in Figure 4. One version of the skip connection layer only has one convolutional layer, which is designed as 1 × 1 convolutional kernels to change the number of channels. The structure of this version is shown in Figure 4. The other version of the skip connection layer has both convolutional layer and batch normalization layer, the configuration of convolutional layer is the same as the former. The structure of our choosen version is shown in Figure 4. In our research, the skip connection layer increased the batch normalization layer, which promoted the quality of classification effectively for thyroid nodules. Specifically, we conducted a comparative experiment to prove this viewpoint.

### 3.5. HAC Block

In order to reduce the loss of spatial information caused by downsampling, we employed dilated convolution substitute downsampling in the model (Liu et al., 2017). Besides, dilated convolution can increase receptive field size, even will not reduce the spatial resolution of the intermediate feature map. The dilated convolution can be described as follows:


(1) $$\begin{array}{l} Y\left(p\right)=\overset{n}{\underset{0}{\sum }}F_{i}\left(p+r\times n\right) \end{array}$$

where *Y*() is the output feature map, *F*_*i*_() is the input feature map, *p* is the processing pixel, *n* is the pixel used in the convolution process, and *r* is the dilation rate. The dilation rate depends on the stride of the input feature map. The dilated convolution is commonly available in two connection of types called parallel type and cascade type (Xiong et al., 2021). The HAC has parallel mode and cascade mode. In a word, the output feature map consists of four aisles atrous convolution and the operation of dimension mapping. Specifically, the output signal of the HAC block is defined as:


(2) $$\begin{array}{l} H=A_{c1}\left(F_{i}\right)∥\left\{\left[A_{c2}\left(F_{i}\right)∥A_{c3}\left(F_{i}\right)∥A_{c4}\left(F_{i}\right)\right]∥D\left(F_{i}\right)\right\} \end{array}$$

(3) $$\begin{array}{l} \{\begin{array}{l} A_{c1}\left(F_{i}\right)=A_{r1}\left(F_{i}\right),\\ A_{c2}\left(F_{i}\right)=A_{r3}\left(F_{i}\right),\\ A_{c3}\left(F_{i}\right)=A_{r1}\left(F_{i}\right)+A_{r3}\left(F_{i}\right),\\ A_{c4}\left(F_{i}\right)=A_{r1}\left(F_{i}\right)+A_{r3}\left(F_{i}\right)+A_{r7}\left(F_{i}\right), \end{array} \end{array}$$

**Figure 5:**
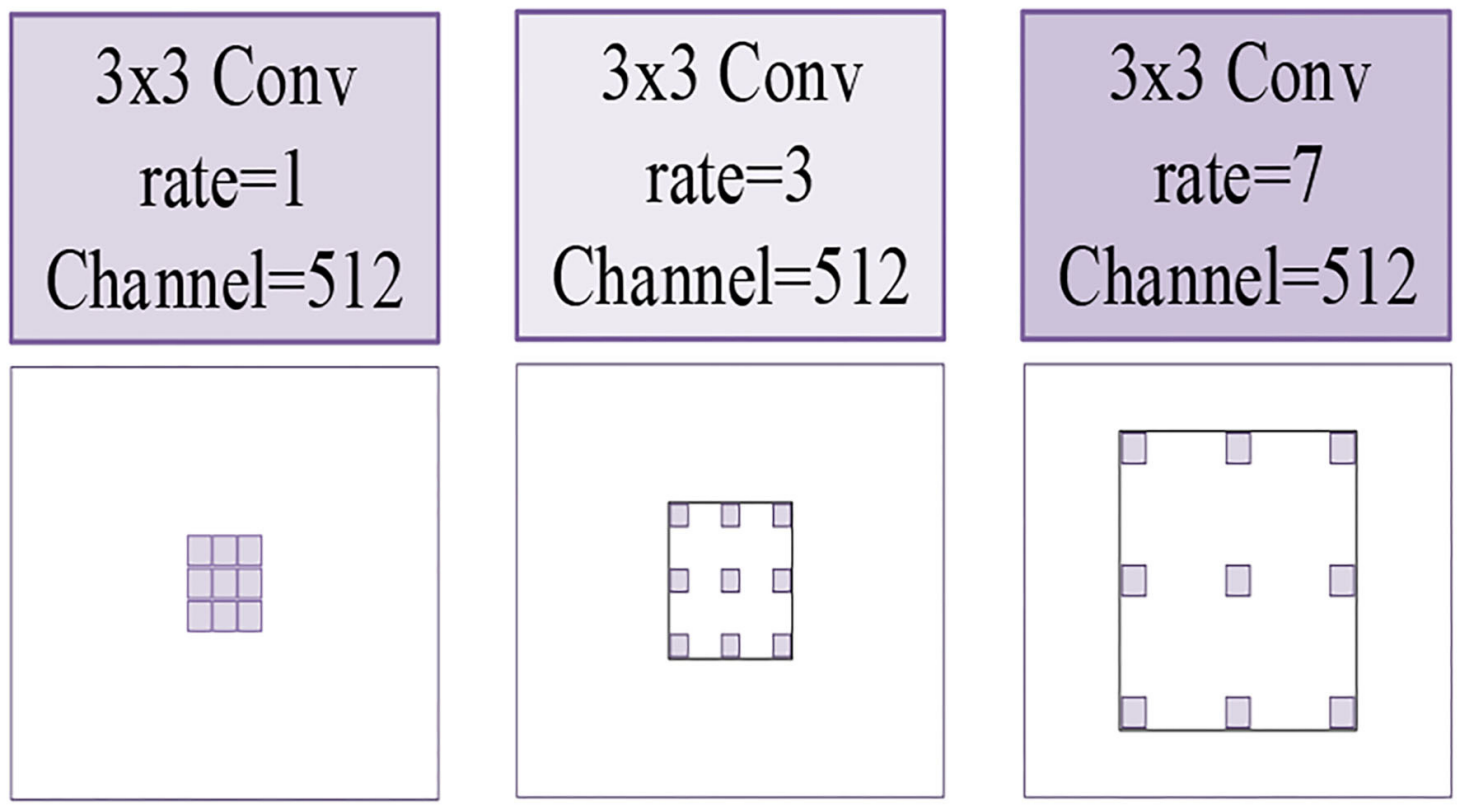
The illustrations of three kinds of atrous convolutions. Left to right: the atrous convolution have dilation rates of *r* = 1, 3, 7, respectively.

**Figure 6:**
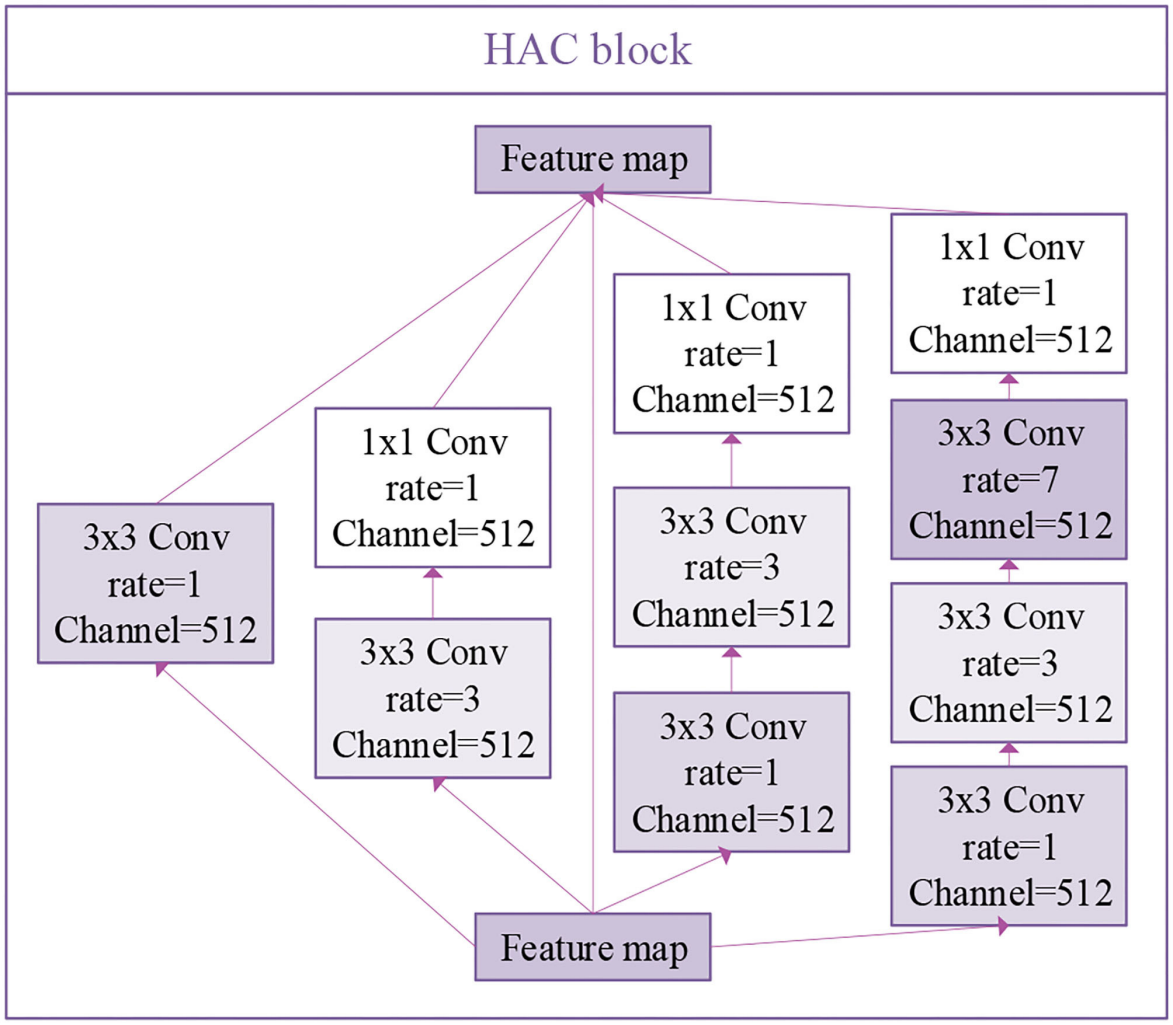
The architecture of HAC with four aisles atrous convolution and the operation of dimension mapping (see the part of white).

In our HAC block, we adopt three atrous convolutions. The architecture of these three atrous convolutions is shown in Figure 5. Due to the fact that we choose atrous convolution with four aisles, where *A*_*c*1_(*F*_*i*_) is the first aisle for atrous convolution, *A*_*r*1_(*F*_*i*_), *A*_*r*3_(*F*_*i*_), and *A*_*r*7_(*F*_*i*_) is atrous convolution with a learning rate of 1, 3, 7, respectively, *H* is the output feature map, and *D*(*F*_*i*_) is the operation of dimension mapping. Then, we display each aisle atrous convolution in particular, as shown in Figure 6. This framework with HAC effectively enlarges the receptive fields of the network to aggregate global information. In our research, the HAC block with parallel mode and cascade mode have evidently helpful improvement in classification ACC. In the experimental part, we compared the HAC block with the atrous spatial pyramid pooling (ASPP) block to verify the superiority of the HAC block in improving the classification accuracy of thyroid nodules.

## 4. Experiments

### 4.1. Datasets

We employ 2,615 ultrasound thyroid nodule images that were manually labeled by doctors in Fujian Union Medical College Hospital, called the TNUI-2021 dataset, which evaluates the robustness and effectiveness of our classification method. There are 1,834 training samples, 523 validation samples, and 258 testing samples. In addition, the original image size of the TNUI-2021 dataset is 780 × 780, we resize the images to 224 × 224 in the image preprocessing stage.

### 4.2. Implementation Details

All models in this experiment were trained on an Ubuntu system with Nvidia RTX 2080TI GPUs. The experiments were performed with SGD as the optimizer, CrossEntropyLoss as the loss, initial learning rate set to 0.1, and weight decay set to 0.001. Two hundred epochs were performed for all experiments.

### 4.3. Evaluation Metrics

In order to evaluate the classification performance, several model evaluation indices are used in the experiment, including ACC, Average Precision (AP), area under the receiver operator curve (AUC), Precision, F1-score, and Specificity, which are calculated as follows:


(4) $$\begin{array}{l} ACC=\frac{TP+TN}{TP+TN+FP+FN}, \end{array}$$

(5) $$\begin{array}{l} Precision=\frac{TP}{TP+FP}, \end{array}$$

(6) $$\begin{array}{l} Specificity=\frac{TN}{TN+FP}, \end{array}$$

(7) $$\begin{array}{l} Recall=\frac{TP}{TP+FN}, \end{array}$$

(8) $$\begin{array}{l} F1-score=\frac{2}{\frac{1}{Precision}+\frac{1}{Recall}}, \end{array}$$

(9) $$\begin{array}{l} AUC=\frac{\sum_{i\in positive\;class}rank_{i}-\frac{M\times \left(M+1\right)}{2}}{M\times N}, \end{array}$$

(10) $$\begin{array}{l} AP=\underset{j}{\sum }\left(Recall_{j}-Recall_{j-1}\right)\times Precision_{j} \end{array}$$

where *TP*, *TN*, *FP*, and *FN* denote the number of true positives, true negatives, false positives, and false negatives, respectively. *M* is the number of positive samples. *N* is the number of negative samples. *rank*_*i*_ is the serial number of the i-th sample. The ACC displays the performance of our n-ClsNet model in classifying nodules as malignant or benign. Specificity shows the proportion of correctly identified benign nodules (Wang et al., 2018).

### 4.4. Method Comparison

**Figure 7:**
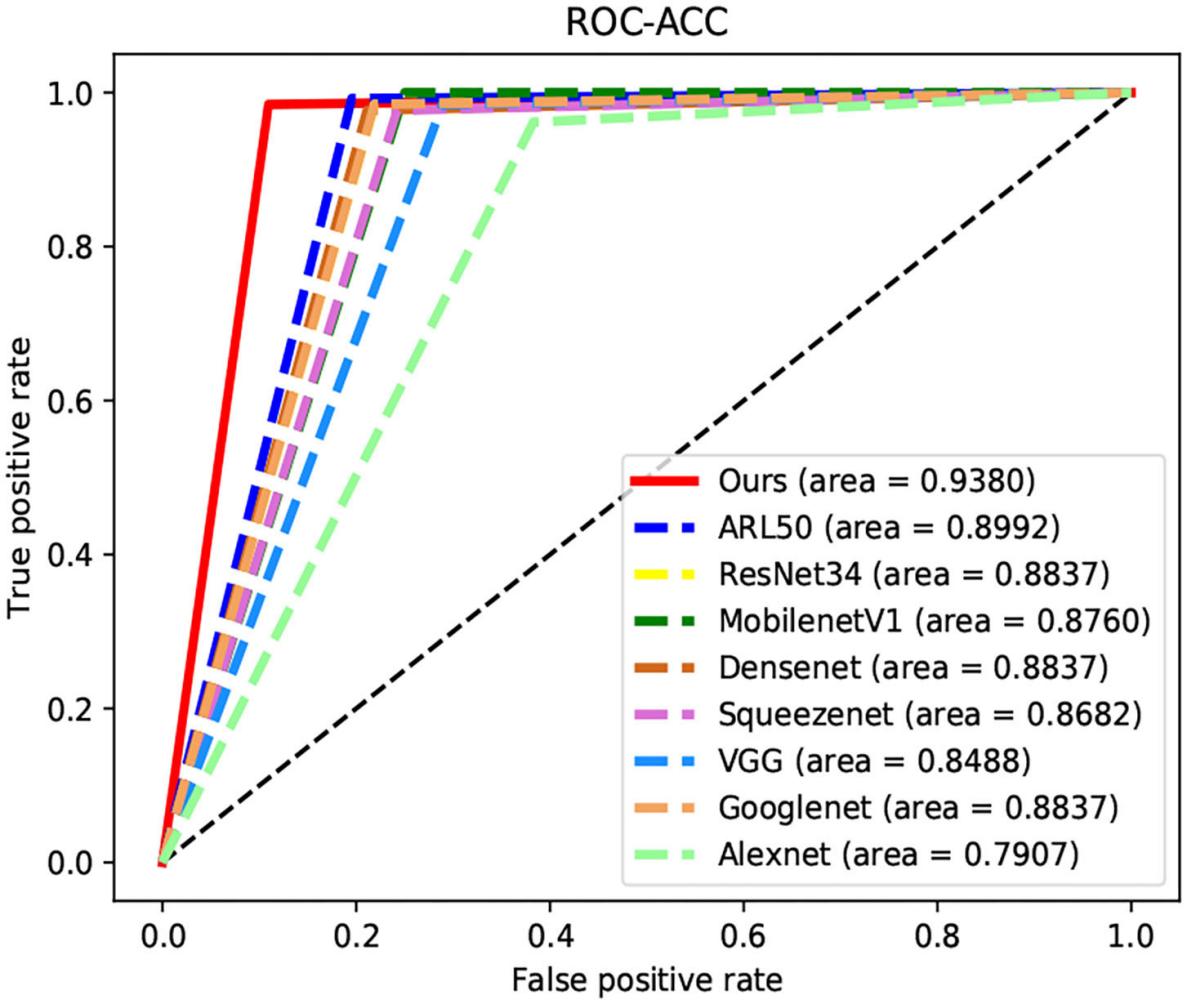
Comparison of receiver operator curve (ROC)-Accuracy (ACC) curves of nine classification approaches on TNUI-2021 datasets.

We compare our proposed model with several representative state-of-the-art classification approaches on the TNUI-2021 dataset from the comparison shown in Table 1. We compare the proposed n-ClsNet with the state-of-the-art classification algorithms ARL50 (Zhang et al., 2019b) used for medical imaging. In addition, some classical deep learning based classification methods, Alexnet (Krizhevsky et al., 2012), GoogleNet (Szegedy et al., 2015), VGG (Simonyan and Zisserman, 2014), ResNet34 (He et al., 2016), SqueezeNet (Iandola et al., 2016), MobilenetV1 (Howard et al., 2017), and DenseNet (Huang et al., 2017), are also included in the comparison. In order to explain intuitively, we compare of receiver operator curve (ROC)-Accuracy (ACC) curves of nine classification approaches on TNUI-2021 datasets, as shown in Figure 7.

**Table 1:** Performance of our method and other methods in classification of thyroid nodules.

**Method**	**ACC**	**AP**	**AUC**	**Precision**	**Specificity**
ARL50 (Zhang et al., 2019b)	0.8992	0.9090	0.9343	0.8377	0.8047
ResNet34 (He et al., 2016)	0.8837	0.8638	0.9218	0.8205	0.7813
MobilenetV1 (Howard et al., 2017)	0.8760	0.8474	0.8852	0.8025	0.7500
DenseNet (Huang et al., 2017)	0.8837	0.9555	0.9607	0.8247	0.7891
SqueezeNet (Iandola et al., 2016)	0.8682	0.9286	0.9375	0.8038	0.7578
VGG (Simonyan and Zisserman, 2014)	0.8488	0.9179	0.9266	0.7758	0.7109
GoogleNet (Szegedy et al., 2015)	0.8837	0.9290	0.9439	0.8205	0.7813
Alexnet (Krizhevsky et al., 2012)	0.7907	0.7553	0.8096	0.7184	0.6172
Ours	**0.9380**	**0.9738**	**0.9756**	**0.9014**	**0.8906**

*Bold values indicate optimal values*.

On comparison with the ARL50 (Zhang et al., 2019b), which is attention residual learning convolutional neural network, the *ACC* increases from 0.8992 to 0.938, the *AP* increases from 0.909 to 0.9738, the *AUC* increases from 0.9343 to 0.9756, the *Precision* increases from 0.8377 to 0.9014, and the *Specificity* increases from 0.8047 to 0.8906. For classic algorithms, compared with ResNet34 (He et al., 2016), the *ACC* is increased by 5.4% from 0.8837 to 0.938, the *AP* is increased by 11% from 0.8638 to 0.9738, the *AUC* is increased by 5.3% from 0.9218 to 0.9756, the *Precision* is increased by 8% from 0.8205 to 0.9014, and the *Specificity* is increased by 10% from 0.7813 to 0.8906, respectively. We also compare n-ClsNet with the MobilenetV1 Howard et al. (2017), the *ACC* increases from 0.876 to 0.938 by 6.2%, the *AP* increases from 0.8474 to 0.9738 by 12%, the *AUC* increases from 0.8852 to 0.9756 by 9%, the *Precision* increases from 0.8025 to 0.9014 by 9%, and the *Specificity* increases from 0.75 to 0.8906 by 14%. For the model evaluation index *ACC*, compared with DenseNet (Huang et al., 2017), the *ACC* increases from 0.8837 to 0.938; compared with SqueezeNet (Iandola et al., 2016), the *ACC* increases from 0.8682 to 0.938; compared with VGG (Simonyan and Zisserman, 2014), the *ACC* increases from 0.8488 to 0.938; compared with GoogleNet (Szegedy et al., 2015), the *ACC* increases from 0.8837 to 0.938; compared with Alexnet (Krizhevsky et al., 2012), the *ACC* increases from 0.7907 to 0.938.

On the thyroid nodule dataset, our model achieves a remarkably higher *ACC*, *AP*, *AUC*, *Precision*, and *Specificity* than others, the highest *ACC* of 0.938, the highest *AP* of 0.9738, the highest *AUC* of 0.9756, the highest *Precision* of 0.9014, and the highest *Specificity* of 0.8906, which proves that our proposed method has a robustness classification ability.

### 4.5. Comparison of Skip-Block With Residual Architecture

#### 4.5.1. Comparative Experiment for the ResNet34-Residual

To verify the effectiveness of skip-block, we conducted an experiment that skip-block has better performance, in contrast with the residual architecture from ResNet34 (He et al., 2016). A quantitative comparison is shown in Table 2. The “No-Batch-Normalization” is one of the versions of skip-block we designed, which has only one convolution layer. The table shows that “No-Batch-Normalization” surpasses “ResNet34-Residual” in the model evaluation of *ACC*, *AP*, *F*1−*score*, *Precision*, and *Recall*. From the comparison, our final version skip-block achieves 0.9341, 0.9726, 0.9337, 0.8844, and 0.9336 in *ACC*, *AP*, *F*1−*score*, *Precision*, and *Recall*, respectively, better than “ResNet34-Residual.”

**Table 2:** Comparison to skip-block and residual architecture.

**Method**	**ACC**	**AP**	**F1-score**	**Precision**	**Recall**
ResNet34-Residual	0.9109	0.9224	0.9104	0.8639	0.9103
No-BatchNormalization	0.9341	0.9726	0.9337	0.8844	0.9336
Ours	**0.9380**	**0.9738**	**0.9378**	**0.9014**	**0.9376**

*Bold values indicate optimal values*.

#### 4.5.2. Comparative Experiment for Two Versions of Skip-Block

In order to further improve our n-ClsNet classification network ACC of thyroid nodules, we consider that the batch normalization layer possesses the advantage of reducing the risk of overfitting and mitigating the disappearance of gradients. We also tried adding a batch normalization layer in the skip connection layer to improve network performance. Experiments show that this attempt is successful. In comparison with the “No-Batch-Normalization,” the *ACC* increases from 0.9341 to 0.938, the *AP* increases from 0.9726 to 0.9738, the *F*1−*score* increases from 0.9337 to 0.9378, the *Recall* increases from 0.9336 to 0.9376, and the *Precision* increases from 0.8844 to 0.9014 by 1.7%. The *Precision* score of ours is significantly beyond “No-Batch-Normalization” architecture, which shows that our proposed final version skip-block is beneficial for thyroid nodules classification.

### 4.6. Comparison of HAC Block With ASPP Block

To verify the superiority of the HAC block compared to the ASPP block, we conducted a comparative experiment. According to the principle of the control variable method, in the experiments, we only replaced the HAC block with the ASPP block. As shown in Table 3, when comparing our employed HAC block to the ASPP block, the *ACC* increases from 0.9186 to 0.938, the *Specificity* increases from 0.8593 to 0.8906, the *F*1−*score* increases from 0.9182 to 0.9378, the *Precision* increases from 0.8758 to 0.9014, and the *Recall* increases from 0.9181 to 0.9376. From the comparison, the classification ACC of our HAC block is much higher than that of the ASPP block.

**Table 3:** Comparison to HAC block and the atrous spatial pyramid pooling (ASPP) block.

**Method**	**ACC**	**Specificity**	**F1-score**	**Precision**	**Recall**
ASPP	0.9186	0.8593	0.9182	0.8758	0.9181
Ours	**0.9380**	**0.8906**	**0.9378**	**0.9014**	**0.9376**

*Bold values indicate optimal values*.

### 4.7. Ablation Study

To evaluate the utility of the multi-scale classification layer, skip-block, and HAC block in our deep learning model, control variable comparison experiment is shown in Table 4. Afterward, we perform the ablation studies using the TNUI-2021 dataset as examples:

**Table 4:** Ablations study for each component of our n-ClsNet framework on the TNUI-2021 dataset.

**Method**	**ACC**	**AP**	**F1-score**	**Precision**	**Recall**
SkipBlock+HAC	0.8760	0.9128	0.8738	0.8025	0.8750
SkipBlock+Multiscale	0.9147	0.9614	0.9141	0.8600	0.9141
SkipBlock+Multiscale+HAC	**0.9380**	**0.9738**	**0.9378**	**0.9014**	**0.9376**

*Bold values indicate optimal values*.

#### 4.7.1. Ablation Study for Employing Multi-Scale Classification Layer

We adopted the multi-scale classification layer to obtain multi-scale feature maps to improve the learning ability. As we can see from the “SkipBlock+HAC,” the evaluation score of *ACC*, *AP*, *F*1−*score*, *Precision*, and *Recall* have been significantly improved: the *ACC* is increased by 6.2% from 0.876 to 0.938, the *AP* is increased by 6.1% from 0.9128 to 0.9738, the *F*1−*score* is increased by 6.4% from 0.8738 to 0.9378, the *Precision* is increased by 9.8% from 0.8025 to 0.9014, and the *Recall* is increased by 6% from 0.875 to 0.9376, respectively. Therefore, the results demonstrate that the multi-scale classification layer is effective.

#### 4.7.2. Ablation Study for Adopting the HAC

We employed the hybrid atrous convolution substitute downsampling, aiming at increasing receptive field size. As shown in Table 4, our selected HAC block improves the *ACC*, *AP*, *F*1−*score*, *Precision*, and *Recall* in thyroid nodules classification than “SkipBlock+Multiscale”: the *ACC* increases from 0.9147 to 0.938, the *AP* increases from 0.9614 to 0.9738 by 2.3%, the *F*1−*score* increases from 0.9141 to 0.9378 by 2.3%, the *Precision* increases from 0.86 to 0.9014 by 4.1%, and the *Recall* increases from 0.9141 to 0.9376 by 2.3%. Even though the evaluation score of *AP* is already performed very well, it has also improved. It demonstrates that our HAC block is useful for the classification task.

## 5. Conclusion

We present a multi-scale deep learning model, namely n-ClsNet, to classify benign and malignant thyroid nodules on ultrasound images, which use multi-scale ultrasound images as input. On the one hand, our skip-block adopts the strategy of approximate jump connection to excavate the feature of the thyroid nodule image. On the other hand, we propose the HAC that takes the place of downpooling to increase receptive field size and decrease the spatial resolution of the intermediate feature map. The experimental results demonstrate that the n-ClsNet model can effectively improve the performance of classification in thyroid nodules. Moreover, our method surpass the performance of representative state-of-the-art classification methods in the thyroid nodules classification task.

## Data Availability Statement

The raw data supporting the conclusions of this article will be made available by the authors, without undue reservation.

## Author Contributions

LW, XZ, XN, XL, JL, HZ, QG, MD, TT, EX, and MZ: concept and design. LW, XZ, XN, XL, EX, SC, CC, QG, TT, and MZ: acquisition of data. LW, XZ, XN, XL, QG, and TT: model design. LW, XZ, XN, XL, TT, QG, and MZ: data analysis. LW, XZ, XN, XL, EX, TT, QG, and MZ: manuscript drafting. LW, XZ, XN, XL, JL, HZ, SC, CC, QG, MD, TT, EX, and MZ: approval. All authors contributed to the article and approved the submitted version.

## Funding

This work was supported by the National Natural Science Foundation of China under grant nos. 61901120 and 62171133, sponsored by Fujian provincial health technology project (2019-1-33), in part by the Science and Technology Innovation Joint Fund Program of Fujian Province of China under grant no. 2019Y9104.

## Conflict of Interest

The authors declare that the research was conducted in the absence of any commercial or financial relationships that could be construed as a potential conflict of interest.

## Publisher's Note

All claims expressed in this article are solely those of the authors and do not necessarily represent those of their affiliated organizations, or those of the publisher, the editors and the reviewers. Any product that may be evaluated in this article, or claim that may be made by its manufacturer, is not guaranteed or endorsed by the publisher.
